# Microstructural Evolution of Diamond-Based Composites at High Temperature and High Pressure

**DOI:** 10.3390/ma15248753

**Published:** 2022-12-08

**Authors:** Tianxu Qiu, Jianwei Feng, Bo Cai, Guojiang Fan, Wei Zhang, Yong Liu

**Affiliations:** 1 State Key Laboratory of Powder Metallurgy, Central South University, Changsha 410083, China; 2 Sino Powder (Henan) Superhard Materials Co., Ltd., Zhengzhou 450001, China

**Keywords:** multi-element alloy, PCD, HTHP, diamond skeleton, thermodynamic

## Abstract

Improving the toughness of diamond composites has become an industrial demand. In this work, Co_50_Ni_40_Fe_10_ multi-element alloy was designed as binder for diamond-based composites prepared by high temperature and high pressure (HTHP). Two methods of mixing-sintering and infiltration-sintering were used to prepare diamond-based composites with different diamond contents. The phase diagrams of Co-C and Co_50_Ni_40_Fe_10_-C at 6 GPa were calculated by Thermo-Calc. The results show that Co_50_Ni_40_Fe_10_ multi-element alloy promotes the sintering of diamond powder than element Co. The transverse rupture strength (TRS) of sintered diamond with Co_50_Ni_40_Fe_10_ (Co_50_Ni_40_Fe_10_-75 vol% diamond) is higher than that of Co-Comp (Co-75 vol% diamond). The TRS of polycrystalline diamond (PCD) with Co_50_Ni_40_Fe_10_ alloy binder is up to 1360.3 MPa, which is 19.2% higher than Co-PCD. Compared with Co, using Co_50_Ni_40_Fe_10_ as binder results in a less metal residue in PCD, while the metal cluster area is smaller and the metal distribution is more uniform.

## 1. Introduction

Diamond has the highest hardness and thermal conductivity, good semiconductor properties and excellent corrosion resistance [1,2,3]. Synthesis of high-performance diamond has been widely studied. Tian et al. [4,5,6] used onion carbon nanoparticles as the precursor to synthesize nano-twin diamonds with an average twin thickness of ~5 nm at superhigh temperature and pressure. The nano-twin diamonds have higher hardness and oxidation resistance than natural diamonds. Liu et al. [7] explored the temperature-pressure reaction of amorphous carbon formed by heating fullerenes at pressure close to the cage collapse boundary (about 20–37 GPa). Millimeter scale nearly full sp3 amorphous carbon block materials with high hardness, elastic modulus and thermal conductivity were successfully synthesized. The studies provide new ideas for the artificial synthesis of superhard materials, but it is difficult to be commercially applied in a short time due to the harsh synthesis conditions.

Diamond is being widely used in cutting tools, drilling tools and abrasive tools for processing and drilling industries [8,9,10,11]. However, the inherent brittleness of diamond seriously restricts the service life of diamond tools. The toughness of polycrystalline diamond (PCD) is better than that of single crystal diamond [12], but it may not be able to meet the requirements of complex working conditions in some industrial applications. Traditional PCD is prepared from a layer of diamond powder on cemented carbide billet at high temperature and high temperature (HTHP). Co in cemented carbide melts and infiltrates into the diamond powder layer, catalyzes the formation of covalent bond between diamond particles, and finally polycrystalline diamond was bonded together with cemented carbide [13]. The polycrystalline diamond layer and the cemented carbide substrate are collectively called polycrystalline diamond compact (PDC). There are some studies on improving the performance of diamond based composite products. Li et al. [14] directly synthesized polycrystalline diamond at 16 GPa and 2573 K without catalyst, which shows extremely high hardness and wear resistance. Although the material harder than natural diamond can be synthesized at ultra-high pressure, the high equipment cost hinders its commercial application. How to synthesize polycrystalline diamond with better mechanical properties at relatively low pressure and temperature is of more significance for the industrial applications.

At the pressure of 5–7 GPa, the rate of covalent bond formation between diamond particles is very slow [15,16]. Elements in Group VIII play an important role in transporting C atoms and catalyzing the formation of sp3 bonds between C atoms in the sintering process [17,18]. Therefore, it is of great significance to design new binders to improve the bonding strength of diamond skeleton in PCD. Meanwhile, the binder (usually Co) may also catalyze the conversion of diamond to graphite at low pressure and high temperature [19,20]. Similarly, diamond tools cannot be used to machine ferrous metals. Removing Co from PCD is a good method to improve the service life of products [21]. Jia et al. [22] prepared PDC at HTHP with Fe_55_Ni_29_Co_16_ alloy as the sintering solvent. It was found that the residual stress of PCD layer is smaller due to the low viscosity and high soakage capability of the binder. This shows that the distribution of binder in PCD has an important influence on the mechanical properties of the composite [23,24].

Taken together, the binder metal plays an important role in the synthesis and the application of PCD. The composition and properties of Co based multi-element alloys can be regulated and controlled in a wide range [25], with a lower melting point, higher strength and toughness than pure Co. In this work, Co_50_Ni_40_Fe_10_ multi-element alloy was designed as the binder to prepare diamond-based composites at HTHP. The thermodynamics of Co-C and Co_50_Ni_40_Fe_10_-C at 6 GPa were studied. The binder in PCD with high diamond content is trapped inside, and it is difficult to completely remove the binder. Therefore, two methods of mixing-sintering and infiltration-sintering were used to prepare diamond-based composites with different diamond contents. Metals in composites with low diamond contents are easy to remove due to the interconnection of the binders. In addition, it is convenient to study the strength of the diamond skeleton. Therefore, the microstructures and mechanical properties of diamond-based composites and diamond skeleton were studied.

## 2. Materials and Methods

**Figure 1:**
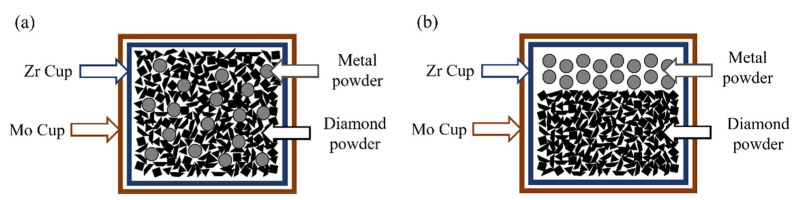
Schematic of powder assembly: (**a**) mixing-sintering method; (**b**) infiltration-sintering method.

Two particle sizes of diamond powder were used in this work, 62~75 μm and 7~15 μm, respectively. Meanwhile, Co_50_Ni_40_Fe_10_ (at%) alloy powder with a particle size of ~15 μm and Co powder with a particle size of 1~2 μm were used. Composites with 75 vol% diamond (62~75 μm) were prepared by mixing-sintering method (Figure 1a). Low content of rough diamond is convenient to the binder removal. It is possible to obtain the diamond skeleton with completely removed binder. The preparation process of the mixing-sintering method included: mechanically mixing 25 vol% metal powder and 75 vol% diamond powder for 20 h, and then sintered at 5.0~5.5 GPa and 1573~1673 K for 16.5 min to obtain diamond composites. Co_50_Ni_40_Fe_10_-75 vol% diamond and Co-75 vol% diamond composites are named Co_50_Ni_40_Fe_10_-Comp and Co-Comp, respectively. The infiltration-sintering method (Figure 1b) was used to prepare PCD with high diamond contents. In order to obtain high-performance PCD, finer diamond powder (7~15 μm) was used, and the synthesis pressure was also increased. In this method, a layer of metal powder was laid on the diamond powder and assembled in the synthesis device, and then sintered at 6.0~6.5 GPa and 1573~1673 K for 16.5 min. The polycrystalline diamonds with Co_50_Ni_40_Fe_10_ and Co as binders are named Co_50_Ni_40_Fe_10_-PCD and Co-PCD, respectively. The HTHP synthesis process was to increase the pressure first, and then the temperature. After synthesis, the pressure was reduced when the temperature dropped below 573 K.

The scanning electron microscope (Quanta FEG 250, FEI Company, Hillsboro, OR, USA) equipped with Energy Dispersive Spectroscopy (EDS) was used to observe the microstructures and analyze the composition of the composites. The energy spectrum analyses were carried out at least three times to obtain the average value. The D/max 2550 X-ray diffractometer (Rigaku Corporation, Akishima, Japan) was used for phase analysis (voltage: 40 kV, current: 450 mA, target material: Cu, step size: 0.02°, scanning speed: 8°/min). A laser was used to cut the composites to obtain a sample size of 16.5 mm × 5.0 mm × 4.0 mm. Aqua regia was used to remove the metal binder in composites to obtain diamond skeleton. The transverse fracture strength (TRS) of composites and diamond skeletons was measured by electronic testing machine (Type 3369, Instron Corporation, Norwood, MA, USA), with a span of 13.0 mm and a loading speed of 1.0 mm/min. Three valid samples were tested for each material. The melting point of Co_50_Ni_40_Fe_10_ alloy was measured in argon atmosphere by high temperature differential scanning calorimeter (DSC 404 F3 Pegasus, Netzsch, Selb, Germany), and the heating rate was 20 °C/min. The phase diagrams of Co-C and Co_50_Ni_40_Fe_10_-C at 6 GPa were calculated by Thermo-Calc 3.0.1, and TCFE7 was selected as the database.

## 3. Results and Discussion

### 3.1. Microstructures

**Figure 2:**
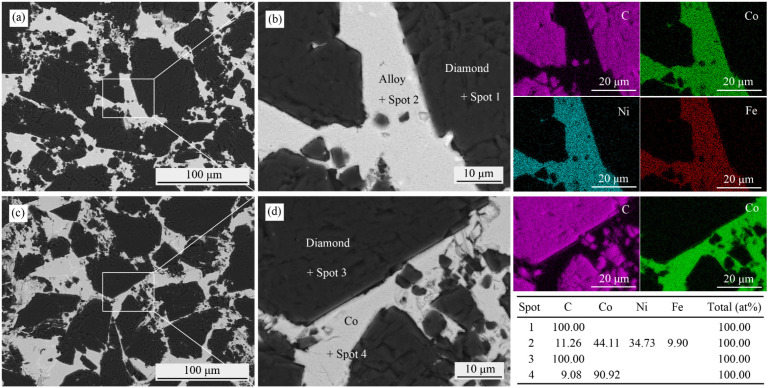
Microstructures, elemental distributions of Co_50_Ni_40_Fe_10_-Comp (**a**,**b**) and Co-Comp (**c**,**d**).

The microstructures and elemental distributions of Co_50_Ni_40_Fe_10_-Comp and Co-Comp are shown in Figure 2. The black phase is diamond, and the gray phase is metal binder (Co_50_Ni_40_Fe_10_ alloy or Co). The binder is distributed uniformly among diamond particles. EDS results show that Co, Ni, Fe elements in the alloy are uniformly distributed without segregation. The carbon concentrations in Co_50_Ni_40_Fe_10_ alloy (Spot 2) and Co (Spot 4) are 11.26 and 9.08 at%, respectively. Although the content of C cannot be accurately measured by EDS, it indicates that the alloy has higher solubility of C to some extent. The binder and diamond are well combined, and the interface is free of cracks. Co, Ni and Fe are not strong carbide forming elements, and no carbide that will damage the mechanical properties is detected at the interface. The microstructure of diamond composites prepared by Co_50_Ni_40_Fe_10_ alloy and Co as binder is almost the same.

**Figure 3:**
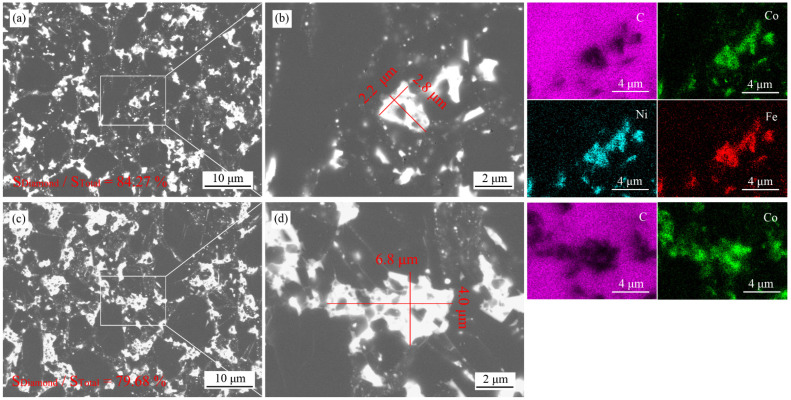
Microstructures, magnified image of metallic cluster and the elemental distributions of Co_50_Ni_40_Fe_10_-PCD (**a**,**b**) and Co-PCD (**c**,**d**).

**Figure 4:**
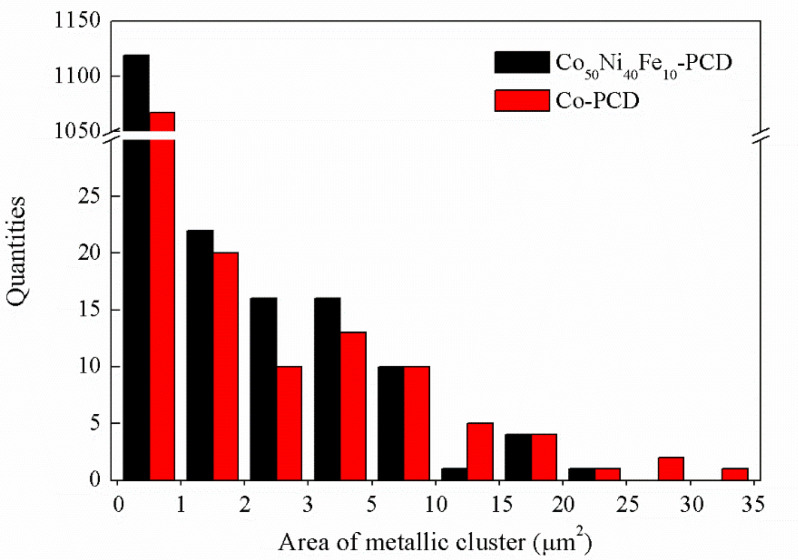
Statistics of area of metallic clusters in Figure 3a,c.

Figure 3 shows the microstructures and elemental distributions of PCD with Co_50_Ni_40_Fe_10_ alloy and Co as binders. It can be seen that the original diamond particles of the two PCDs have been well combined. In the process of HTHP, the alloy melt infiltrates into the gap of diamond particles and catalyzes the formation of C-C bond (sp^3^). With the progress of sintering, the original diamond gradually forms polycrystals, and the alloy melt is discharged from the gap. The nanoscale metal residues in Figure 3b,d show the interface of original diamond particles, representing a good combination between diamond particles. The micron-sized metal residues between original diamond particles is considered harmful to the performance of PCD. The thermal expansion coefficient of metal is one order of magnitude larger than that of diamond. In the process of cutting or drilling, polycrystalline diamond products generate heat by friction. The micro-sized metal residues will generate high thermal stress and lead to failure. Therefore, the area of metal residues in Figure 3a,c was calculated by Image J. According to the statistics shown in Figure 4, there are 3 island shaped metallic clusters of more than 25 μm^2^ in the Co-PCD, and no such island in the Co_50_Ni_40_Fe_10_-PCD. The number of metallic clusters above 10 μm^2^ is 13 in Co-PCD and 6 in Co_50_Ni_40_Fe_10_-PCD. The statistical results of the proportion of diamond phase area in Figure 3a,c show that diamond accounts for 84.27% in Co_50_Ni_40_Fe_10_-PCD and 79.68% in Co-PCD. This shows that compared with Co, Co_50_Ni_40_Fe_10_ alloy has less residue in PCD, and the area of residual metallic cluster is smaller and the distribution of binder is more uniform.

### 3.2. Phase Analyses

**Figure 5:**
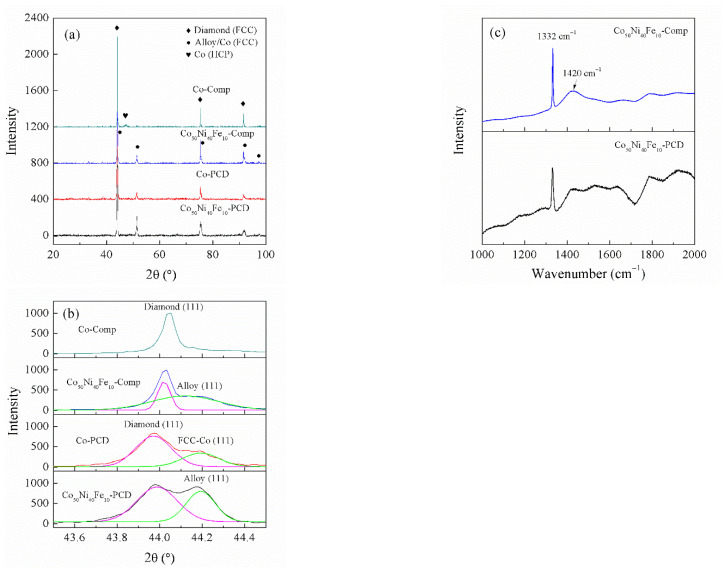
Phase analyses of diamond composites and PCD: (**a**) XRD patterns; (**b**) multi-peaks fitting around 44° in (**a**); (**c**) Raman spectrum of diamond.

The XRD patterns of diamond composites and PCD are displayed in Figure 5a. Peaks of Co_50_Ni_40_Fe_10_ alloy and diamond were detected in Co_50_Ni_40_Fe_10_-Comp and Co_50_Ni_40_Fe_10_-PCD. Diamond and Co_50_Ni_40_Fe_10_ alloy are of FCC structures, and the diffraction peaks of (111), (220) and (311) lattice planes are very close. So, the multi-peaks around 44° were fitted, and the results are shown in Figure 5b. The slight difference in the position and width of diamond (111) peak in PCD and composites may be caused by the difference in raw materials. Due to the large specific surface area of fine diamond it is easy to adsorb impurities, resulting in (111) peak shifting to the left and widening. Raman spectrums of diamond in Co_50_Ni_40_Fe_10_-Comp and Co_50_Ni_40_Fe_10_-PCD are shown in Figure 5c. The characteristic peak of diamond is located at 1332 cm^−1^, and the fluorescence peak near 1420 cm^−1^ is caused by N element doped in diamond. The spectral noise of diamond in PCD is higher because the composite made of fine diamond has more impurities.

In PCD and composites prepared with Co as binder, the crystal structure of Co shows difference. In Co-Comp, it is detected that Co exists in the form of HCP structure, while in Co-PCD, it exists in the form of FCC structure. This is due to the different thermal conductivity of PCD and composites. Diamond exhibits the highest thermal conductivity in nature, while the thermal conductivity of metal is one order of magnitude lower than diamond. The diamond content of the composites (75 vol%) is far lower than that of PCD. It means that the thermal conductivity of the composites is much lower than that of PCD. Therefore, after synthesis, PCD will be cooled at a faster rate, and Co is easier to retain its FCC structure at high temperature. On the contrary, the cooling rate of Co-Comp is relatively slow, and Co is transformed into low-temperature HCP structure. The thermal conductivity can also reflect the covalent bonding of diamond particles. PCD with good covalent bonding has higher thermal conductivity, because there is limited interface hindering the heat conduction. The PCD retained the high temperature FCC structure of Co, reflecting a good covalent bonding between diamond particles.

### 3.3. Mechanical Properties

**Figure 6:**
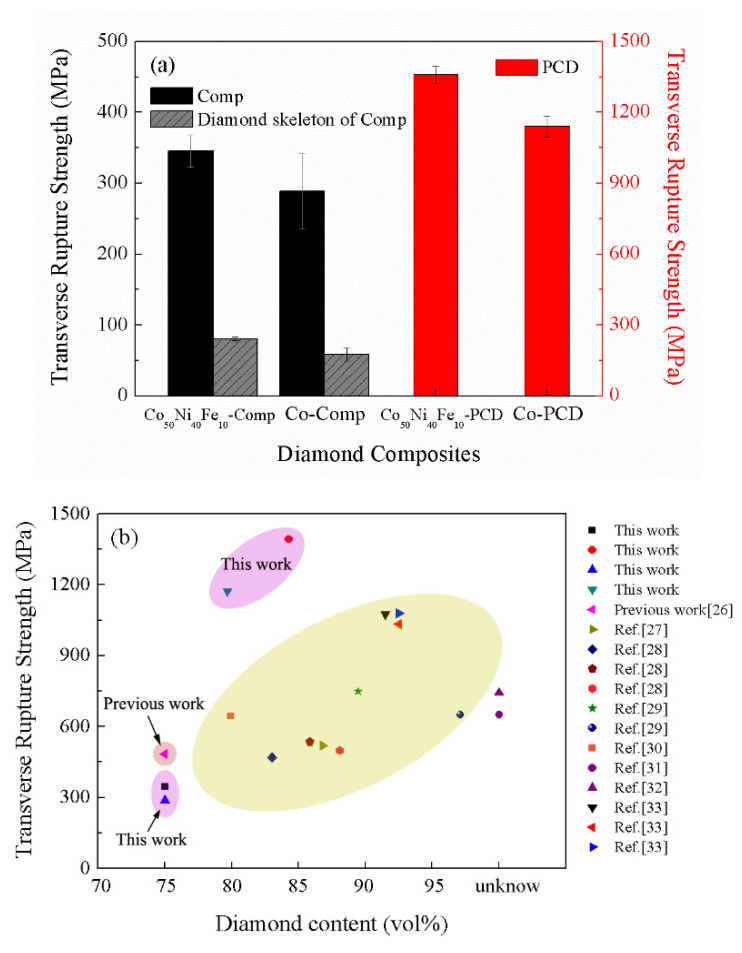
(**a**) Transverse rupture strength of diamond composites, diamond skeleton and PCD; (**b**) Comparison of transverse rupture strength of PCD with different diamond contents in references [26,27,28,29,30,31,32,33].

The values of TRS of diamond composites, diamond skeleton and PCD are shown in Figure 6a. The metallic content in the cross section of diamond skeleton was measured by EDS. The results show that it was lower than 0.5 at%, indicating that the metal binder was almost completely removed. The TRS of Co_50_Ni_40_Fe_10_-Comp (345.5 MPa) is 19.8% higher than Co-Comp (288.4 MPa), and the TRS of diamond skeleton of Co_50_Ni_40_Fe_10_-Comp (80.4 MPa) is 37.9% higher than that of Co-Comp (58.3 MPa). The covalent bonding between diamond grains directly determines the TRS of the diamond skeleton. This illustrates that Co_50_Ni_40_Fe_10_ alloy has better ability to promote the formation of diamond skeleton than Co. For the composites, the diamond content accounts for 75 vol%, its TRS is mainly controlled by the strength of the diamond skeleton. Since the TRS of diamond skeleton of Co_50_Ni_40_Fe_10_-Comp is higher, the TRS of Co_50_Ni_40_Fe_10_-Comp is also higher than that of Co-Comp.

The metal binder in PCD is not connected, so the removal of metal becomes difficult. Long time treatment with aqua regia can only remove the metal on the surface of PCD but cannot remove the metal inside. Therefore, only the mechanical properties of PCD were tested. Due to the high diamond content in PCD, the TRS of PCD is more determined by the bonding strength of diamond skeleton. At the same time, due to the difference in modulus between diamond and metal binder, the large area of metal binder cluster will damage the mechanical properties of PCD. The TRS of Co_50_Ni_40_Fe_10_-PCD is up to 1360.3 MPa, which is 19.2% higher than Co-PCD (1140.7 MPa). The higher TRS of Co_50_Ni_40_Fe_10_-PCD is attributed to the better ability of the alloy to promote the formation of the diamond skeleton, and the uniform distribution of metal binder. The comparisons of TRS between the composites in this work and those in the literature [26,27,28,29,30,31,32,33] are shown in Figure 6b. The diamond content of composites synthesized by HTHP is about 80–95 vol%, and the binders are mainly Co, carbides and ceramics. The TRS of diamond-based composites increased with diamond content. The TRS of PCD prepared by cemented carbide infiltration (commercial products) is about 1000~1100 MPa. The TRS of Co-PCD prepared in this work is a little higher than that of commercial products. At the same time, the TRS of Co_50_Ni_40_Fe_10_-PCD prepared in this work is about 300 MPa higher than that of commercial products.

### 3.4. Fracture Morphologies

**Figure 7:**
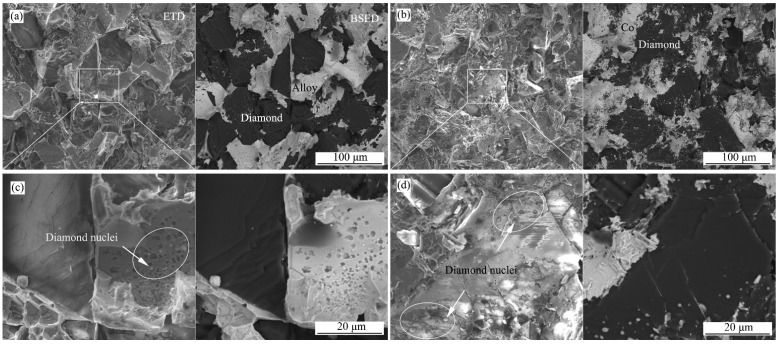
Secondary electron images (EDT, left half) and backscattered electron images (BSED, right half) of fracture morphologies of Co_50_Ni_40_Fe_10_-Comp (**a**,**c**) and Co-Comp (**b**,**d**).

The fracture morphologies of Co_50_Ni_40_Fe_10_-Comp and Co-Comp are shown in Figure 7. Due to the poor conductivity of diamond, the morphologies of diamond and metal binder are improved in the backscattered electron image. Both composites exhibit a brittle trans-granular fracture manner. The metal binder shows some deformation. It is worth noting that on the surface where the diamond grains contact the binder, micron-sized small particles were found. The semi quantitative analyses of EDS show that these particles are the mixtures of metal elements and C. 

The synthesis process of polycrystalline diamond at HTHP is similar to that of conventional powder metallurgy sintering. The binder has two functions in the sintering process, one is to “transport” C atoms, and the other to catalyze the formation of sp^3^ bonds. Therefore, elements in Group VIII can be used as binders, which have both catalytic capacity and solubility for C. The catalytic ability is considered to be closely related to the vacancy numbers of d-orbitals. The number of micron-sized particles in Figure 7c,d reflects the solubility of C in the binder. In the process of high temperature and high pressure sintering, C atoms migrate by dissolution and precipitate in the binder melt. After the sintering process, the material cools rapidly. With the decrease in temperature, the supersaturated C in binder precipitates on the surface of original diamond particles and nucleates heterogeneously to form new diamonds. There are more pits left by secondary precipitation diamond particles in the binder of Co_50_Ni_40_Fe_10_-Comp, reflecting a greater solubility of C in the binder.

**Figure 8:**
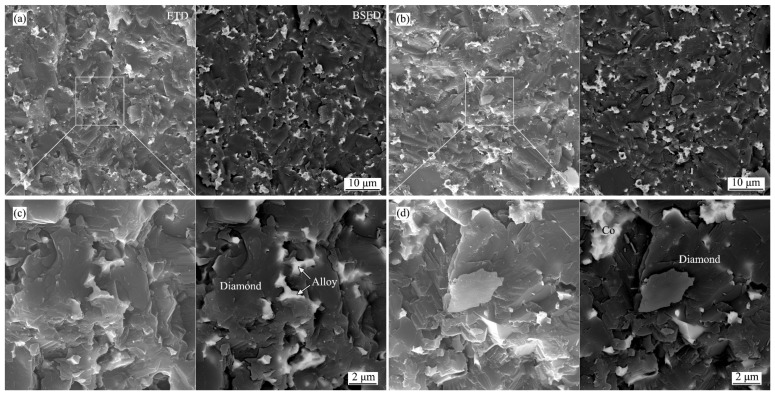
Secondary electron images (EDT, left half) and backscattered electron images (BSED, right half) of fracture morphologies of Co_50_Ni_40_Fe_10_-PCD (**a**,**c**) and Co-PCD (**b**,**d**).

Fracture morphologies of Co_50_Ni_40_Fe_10_-PCD and Co-PCD are shown in Figure 8. The diamond shows cleavage fracture, and the metal binder shows plastic deformation. It shows that the diamond particles formed a good covalent bond connection. Otherwise, the fracture form of polycrystalline diamond should be intergranular fracture. The metal binder and diamond grains are well combined, and there is no crack at the interface. From the backscattered electron image it can be seen that the distribution of metal binder in Co_50_Ni_40_Fe_10_-PCD is more uniform and the cluster area is smaller. The bulk modulus of diamond (443 GPa) is more than twice that of Co, Ni and other metals (about 180–200 GPa). It means that when under loading, the diamond around the large area of metal binder cluster will produce greater stress concentration, eventually leading to failure. Therefore, PCD with less metallic residues and more uniform distribution of metal binder should have higher mechanical properties.

### 3.5. Thermodynamics at HTHP

**Figure 9:**
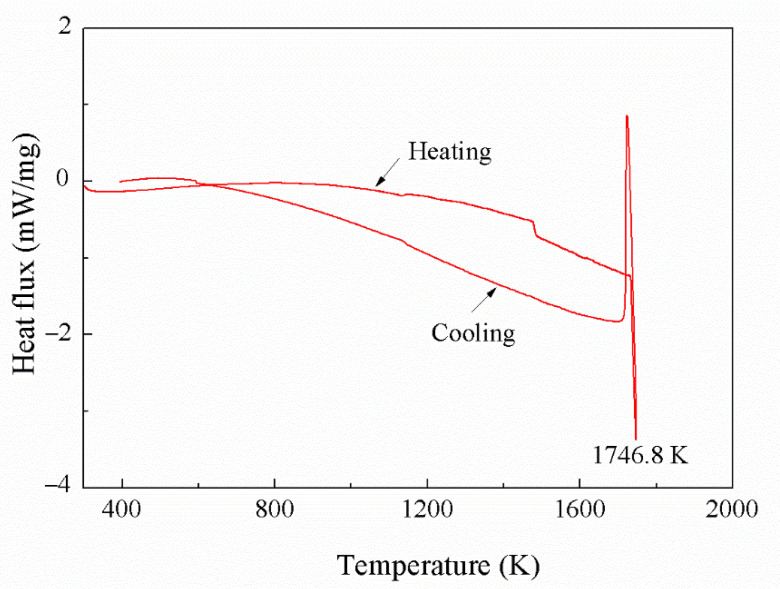
DSC curve of Co_50_Ni_40_Fe_10_ alloy.

**Figure 10:**
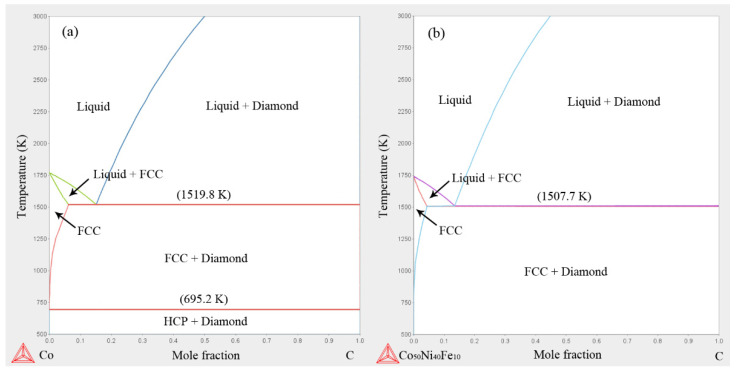
Phase diagrams at 6 GPa calculated by thermo-calc: (**a**) Co-C; (**b**) Co_50_Ni_40_Fe_10_-C.

DSC curve of Co_50_Ni_40_Fe_10_ alloy is shown in Figure 9. It can be seen that the heat flux curve of the alloy is stable during the heating and cooling process, and there are only obvious endothermic and exothermic peaks at the melting point. Combined with XRD analysis, it can be determined that Co_50_Ni_40_Fe_10_ alloy is a homogeneous single-phase FCC structure from room temperature to melting point. The melting point of the alloy measured by DSC is 1746.8 K, which is about 21 K lower than that of Co (1768 K). Phase diagram of Co-C and Co_50_Ni_40_Fe_10_-C at 6 GPa calculated by thermo-calc is shown in Figure 10. The results show that the eutectic point of Co_50_Ni_40_Fe_10_-C is 1507.7 K, which is lower than that of Co-C (1519.8 K). The migration rate of atoms in liquid phase sintering is much higher than that in solid phase sintering. The lower eutectic point indicates that the material system can approach the liquid phase sintering stage earlier, and the sintering is more sufficient at the same conditions.

Sintering process at HTHP can be further determined by phase diagram. In the sintering stage there are alloy liquid phase and diamond phase in the material system. The C atoms in the original diamond particles migrate to the junctions of the particles to form polycrystals driven by the surface energy. The migration of C atoms is carried out by the dissolution and precipitation in liquid phase. At the cooling stage after sintering, supersaturated C atoms in the liquid phase precipitate and transform into diamond. When the temperature drops below the liquidus, the liquid phase of the alloy in the system changes into a solid phase. With the continuous decrease in the temperature, the supersaturated C atoms in the solid alloy are precipitated and transformed into diamond particles of micron size. The calculated results also show that the transition temperature of Co at 6 GPa from FCC structure to HCP structure is 695.2 K. The Co binder in Co-Comp is HCP structure, which exists at low temperature (<695 K). While Co in the PCD prepared in this work is FCC structure, which means that the material retains the phase structure at high temperatures. This illustrates that the PCD has high thermal conductivity, representing a good covalent bond connection between diamond grains. On the other hand, FCC structure has more slip systems than HCP structure, so the plasticity of FCC structured Co is better than that of HCP structure. Better plasticity of binder is beneficial to coordinate deformation and reduce stress concentration.

## 4. Conclusions

Co_50_Ni_40_Fe_10_ multi-element alloy was used as binder in fabricated diamond-based composites at HTHP. The microstructures, mechanical properties and thermodynamics of Co_50_Ni_40_Fe_10_-diamond and Co-diamond composites were investigated. The main findings are summarized as follows:

(1) Co_50_Ni_40_Fe_10_ multi-element alloy promotes the sintering of diamond powder better than element Co, because the melting point of Co_50_Ni_40_Fe_10_ alloy is lower than that of Co;

(2) The TRS of Co_50_Ni_40_Fe_10_-Comp is 19.8% higher than Co-Comp, and the TRS of diamond skeleton of Co_50_Ni_40_Fe_10_-Comp is 37.9% higher than that of Co-Comp, due to the better ability of Co_50_Ni_40_Fe_10_ alloy to promote the formation of diamond-diamond bonding;

(3) The TRS of Co_50_Ni_40_Fe_10_-PCD prepared by the infiltration-sintering method is up to 1360.3 MPa, which is 19.2% higher than Co-PCD. This is attributed to the better ability of Co_50_Ni_40_Fe_10_ alloy to promote diamond sintering by forming diamond-diamond bonding and the uniform distribution of metal binder in the material.

## Data Availability

Not applicable.
